# HINTS+ to diagnose AICA stroke: systematic review of the diagnostic impact of acute unilateral hearing loss in the acute vestibular syndrome

**DOI:** 10.1007/s00415-026-13641-3

**Published:** 2026-02-10

**Authors:** Gitanjali Anburajan, Jorge Kattah, Zheyu Wang, Georgios Mantokoudis, David E. Newman-Toker, Alexander A. Tarnutzer

**Affiliations:** 1 Faculty of Medicine, University of Zurich, Zurich, Switzerland; 2 University of Illinois College of Medicine, Peoria, IL USA; 3 Division of Quantitative Sciences, Sidney Kimmel Comprehensive Cancer Center, Johns Hopkins University School of Medicine, Baltimore, MD USA; 4 Department of Biostatistics, Johns Hopkins Bloomberg School of Public Health, Baltimore, MD USA; 5 Department of Otorhinolaryngology, Head and Neck Surgery, lnselspital, Bern University Hospital, Bern, Switzerland; 6 University of Bern, Bern, Switzerland; 7 Department of Neurology, Johns Hopkins University School of Medicine, Baltimore, MD USA; 8 Department of Epidemiology, Johns Hopkins Bloomberg School of Public Health, Baltimore, MD USA; 9 Neurology, Cantonal Hospital of Baden, 5404 Baden, Switzerland

**Keywords:** Acute vestibular syndrome, Diagnostic accuracy, Acute hearing loss, HINTS-plus, AICA stroke, Head-impulse test

## Abstract

**Purpose:**

Head-Impulse, Nystagmus, Test-of-Skew (HINTS) testing is recommended to identify stroke in the acute vestibular syndrome (AVS). Diagnostic accuracy depends on the vascular territory involved, with lower sensitivity for anterior-inferior cerebellar artery (AICA)- than posterior-inferior cerebellar artery (PICA)-territory strokes. Adding new-onset, acute unilateral hearing loss (AUHL) as a fourth central sign has been proposed (“HINTS+”) to improve sensitivity for AICA-stroke detection.

**Methods:**

We performed a systematic search (MEDLINE, Embase) to identify studies reporting diagnostic accuracy of bedside examination in AVS patients, including the diagnostic impact of HINTS+. Results were stratified by stroke location.

**Results:**

We identified 4388 citations and included 11 articles (*n* = 770 patients). Ischemic strokes (*n* = 319) and acute unilateral vestibulopathy (*n* = 391) were the most frequent causes. AUHL was most often assessed by finger rub (*n* = 311), tuning-fork tests (*n* = 114), or whispered words (*n* = 169), and was present in 50 patients (central = 29, peripheral = 21). Bedside head-impulse testing was abnormal in 25/29 central cases with AUHL, with 19/25 cases demonstrating an AICA-stroke. Diagnostic accuracy of HINTS (sensitivity = 92.7% [88.7–96.8%]; specificity = 91.1% [86.3–95.8%]) was high. Adding AUHL (HINTS+) increased sensitivity (95.7% [92.7–98.7%]) but decreased specificity (80.7% [70.6–90.7%]), though the latter was largely from bias in patient selection. AUHL had the biggest impact for AICA-territory strokes, increasing sensitivity from 73.3% (59.0–84.0%) to 91.1% (79.3–96.5%).

**Conclusions:**

Testing for AUHL in AVS increased stroke-sensitivity, but this effect was mainly related to AICA-strokes with abnormal head-impulse testing (*n* = 19/22), where adding AUHL yielded an absolute increase of 17.8% in stroke-sensitivity. In AVS, AUHL should be assessed at the bedside (and ideally confirmed by audiometry).

**Supplementary Information:**

The online version contains supplementary material available at https://doi.org/10.1007/s00415-026-13641-3.

## Introduction

Representing 2.1–4.4% of all admissions, acute vertigo and dizziness are among the most frequent presenting symptoms to the emergency department (ED) [1, 2]. With 155 million US ED visits annually, this translates to about 5 million consultations per year in the US (and probably 50 to 100 million worldwide [3, 4]), resulting in estimated annual US health care costs of over $10 billion [5]. The differential diagnosis of acute vertigo/dizziness spans many specialties, and the fraction of dangerous, potentially life-threatening causes rises with increasing age [1]. Across all ages, about 15% of patients presenting to the ED with acute dizziness are eventually diagnosed with a sinister underlying cause [1]. Importantly, about 3–5% of all acutely dizzy patients have an underlying ischemic stroke causing their symptoms [3]. This fraction rises to about 25% for the subset of patients presenting with the acute vestibular syndrome (AVS) [6]. ED misdiagnosis appears to be common for vertebrobasilar strokes presenting with dizziness, occurring in nearly 40% of cases [7]. A recent meta-analysis found that about 17% of all cerebrovascular events are missed at initial ED presentation [8], and risks of misdiagnosis rise precipitously when presenting neurologic complaints are mild, transient, or non-specific [7]. For non-specific symptoms, the false negative rate is 39.4% for dizziness/vertigo vs. 4.4% for motor weakness (odds ratio [OR] 14.22 [9.76–20.74]) [7].

Introducing targeted bedside examination techniques focusing on ocular motor and vestibular signs, such as the Head Impulse, Nystagmus, Test-of-Skew (HINTS) in 2009 [9], its expansion HINTS+ in 2013 [10], and the STANDING algorithm in 2015 [11] have substantially improved bedside diagnostic accuracy for detecting stroke in AVS patients [12]. Specifically, the HINTS battery is one of the best clinical tests to differentiate between central and peripheral causes for dizziness. The HINTS battery has a high sensitivity and specificity in detecting central causes of AVS [9]. However, its diagnostic accuracy depends on the integrity of the vestibulo-ocular reflex (VOR), which relates to the vascular territory involved [13, 14]. HINTS sensitivity is lower in strokes affecting the vascular territory of the anterior inferior cerebellar artery (AICA). This is thought to be because of involvement of the labyrinthine artery (usually a distal branch of the AICA), which often leads to labyrinthine infarction and consequently an abnormal head-impulse test (HIT). The HIT in the posterior inferior cerebellar artery (PICA) territory, however, remains predominantly normal, except in rare cases of direct vestibular nucleus damage [15] or labyrinthine involvement (due to variation in the origin of the internal auditory artery or its terminal branch, the labyrinthine artery) [16, 17]. Thus, it has been proposed to include testing for unilateral new-onset hearing loss (AUHL) as a central sign in the HINTS battery. This has been called “HINTS+” [10]. There is good evidence that AICA strokes with positive HIT benefit from a search for AUHL [10], and the rare PICA stroke with abnormal HIT due to labyrinthine infarction likely will as well [16].

This review aimed to assess the diagnostic impact of HINTS+. We assessed whether adding AUHL to HINTS offers an advantage in detecting strokes in AVS patients, and which stroke locations benefit most from this. Furthermore, this review sought to detect possible limitations or pitfalls of HINTS+.

## Materials and methods

### Data sources and searches

We searched MEDLINE and Embase for articles in the English language with terms representing the following three components: (1) vertigo/dizziness, (2) diagnostic accuracy of bedside examination techniques, including assessing hearing loss, and (3) acute vestibular syndrome. We also performed a manual search of reference lists from eligible articles and contacted corresponding authors where necessary. We limited our search to articles published since 2000, when the assessment for subtle ocular motor findings in acute vestibular syndrome first was proposed. Our search was updated through September 23rd, 2024.

### Study selection and quality assessment

Articles were selected by two independent screeners (GA, AAT) using pre-determined inclusion/exclusion criteria and a structured process (see Online Appendix 1 for details). Our focus was on studies examining the diagnostic accuracy of clinical examination techniques for distinguishing between peripheral and central causes of AVS (i.e., symptom onset < 72 h) in patient populations in the ED or on an acute inpatient ward. The AVS was defined as acute and persistent vertigo or dizziness accompanied by nausea/vomiting, motion intolerance, gait unsteadiness, and often nystagmus [18]. The requisite reference standard for “ruling out” stroke in a peripheral vestibular case with high reliability was delayed magnetic resonance imaging with diffusion-weighted images (MRI-DWI); strokes could be “ruled in” using confirmatory neuroimaging, including computed tomography (CT) in the appropriate clinical context, but studies using unconfirmed clinical diagnoses as the predominant reference standard were considered high-risk for bias. Thus, we assessed the overall risk of bias and applicability concerns for all studies selected using QUADAS-2 criteria, however, we did not exclude articles based on their QUADAS-2 rating (such as having a high risk for a patient selection bias due to lacking delayed MRI-DWI in peripheral AVS cases). Full details describing selected studies are provided in supplementary Table 1 in Online Appendix 2. We did not attempt to “re-verify” diagnoses and used diagnostic terminology as applied by source study authors. We recognize that the official definition for acute unilateral vestibulopathy (AUVP) excludes patients with hearing loss [19]. But in the real world/other publications the AUVP may be accompanied by auditory symptoms. We did not expressly seek to rename each one of these cases as either AUVP or labyrinthitis on the basis of the absence/presence of auditory symptoms.

### Data extraction, synthesis and analysis

We report the diagnostic accuracy of clinical examination techniques to distinguish peripheral from central causes of AVS (vestibular eye movement tests, hearing assessment, general neurologic examination). Prospectively defined subgroup analyses were performed, comparing diagnostic test properties stratified by stroke vascular territory (PICA vs. AICA).

Definitions for “obvious” and “subtle” neurologic signs (i.e., based on their likelihood to guide the clinician to the correct diagnosis) in the acutely dizzy were previously published [14]. We adhered to these definitions for this publication. “Subtle” signs such as central-type eye movements or dysmetric saccades were permitted in the isolated central AVS (cAVS) subgroup. Hearing loss was considered a “subtle” sign here as it is commonly believed to indicate a peripheral lesion localization, even though its presence may instead point to a vascular (labyrinthine or lateral pontine infarction) rather than a viral (labyrinthitis) cause of the AVS presentation [10, 17].

We assessed the diagnostic accuracy (using the measures as specified in the next paragraph) for the composite HINTS and HINTS+ test batteries assessing the added value of HINTS+ compared to HINTS overall and for different vascular territories. Specifically, we asked to which extent testing for AUHL improves the diagnostic workup beyond already available test results [20]. For HINTS “peripheral,” the patient needed to have all three features “peripheral” (i.e., Boolean “AND”)—(i) a unilaterally abnormal horizontal HIT with (ii) a direction-fixed, dominantly horizontal nystagmus beating contralaterally to the abnormal horizontal HIT and (iii) without a vertical skew deviation on alternate cover testing. Anything else was considered a “central” HINTS pattern (i.e., Boolean “OR” on any of the “central” findings)—(i) bilaterally normal or bilaterally abnormal horizontal HIT OR (ii) direction-changing gaze-evoked nystagmus or unidirectional horizontal nystagmus beating towards the unilaterally abnormal horizontal HIT or (iii) presence of a vertical skew deviation on alternate cover testing. For HINTS+ “central”, the hearing loss needed to be new-onset, unilateral, and evident by bedside finger rub (or similar bedside testing), device-based testing, or both. We refer to strokes with abnormal HIT as “pseudo-peripheral” to clarify that the HIT finding is counterintuitive; we note that, while brainstem or cerebellar strokes are, in fact, “pseudo,” patients with isolated labyrinthine infarction are, in fact, “peripheral” (even though of dangerous vascular cause). We know that AUHL can occur in either central or peripheral disease, but it is unclear how strong a predictor it is of one or the other. For the purposes of this manuscript, we assessed the diagnostic test properties of AUHL as a sign of stroke/other central diseases.

As measures of diagnostic accuracy, we calculated sensitivity, specificity, negative likelihood ratio (LR−) and positive likelihood ratio (LR+) for ED or acute ward diagnoses for any central vestibular pathology (rather than stroke, per se, since the clinically relevant diagnostic branch point is differentiating central from peripheral disorders). We present proportions and, where appropriate, 95% confidence intervals (95% CI). Summary measures were calculated using a random effects model using the DerSimonian-Laird estimator [21, 22]. Tests of heterogeneity were conducted based on Cochran’s *Q*-test. For a study with zero cells, a continuity correction of 0.5 was added to all cells of that study (not to all studies). Heterogeneity statistics (Cochran’s *Q*-test) were calculated using R v4.2.1 (Foundation for statistical computing, Vienna, Austria) by a PhD biostatistician (ZW). A sensitivity analysis was performed comparing the diagnostic accuracy of HINTS+ for all studies included (*n* = 11) and for a subset of studied (*n* = 9) without two studies with highly selected patient populations [23, 24].

This review is reported in accordance with PRISMA guidelines. The protocol was not registered in PROSPERO. Being a systematic review of the literature, no ethics approval was required.

### Data availability

Source data used for meta-analysis will be made available to others upon request to the corresponding author.

## Results

**Fig. 1:**
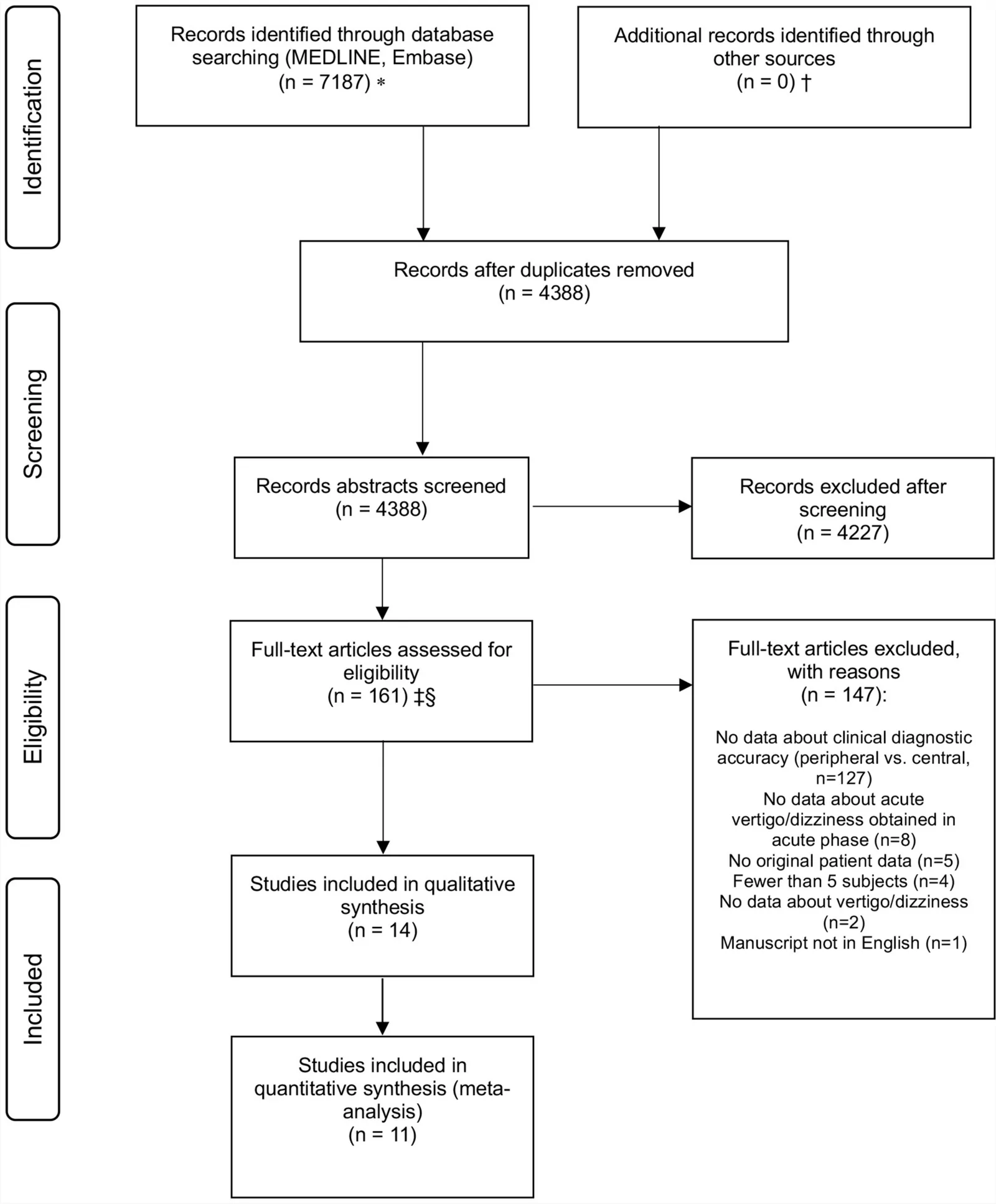
*MEDLINE was accessed via PubMed. ^†^Individual hand search of citation lists from selected studies and investigator files identified 0 additional manuscripts for review. ^‡^Abstracts coded as “yes” or “maybe” by at least one reviewer were included in full-text review. ^§^After full-text evaluation by two reviewers, any differences were resolved by discussion and, if needed, adjudication by a third, independent reviewer

Our search identified 4388 unique citations, of which 4227 (96.3%) were excluded at the abstract level (Fig. 1—PRISMA flow chart). We sought to examine 161 full manuscripts. At the end of our full-text review, 147 manuscripts were excluded and 14 were considered eligible, representing 0.3% of the total (*n* = 4388).

### Study quality assessment using QUADAS-2

Included studies were subjected to a quality assessment using the QUADAS-2 tool. Study quality was heterogeneous. Overall, 5 studies were rated as having a high risk of bias in one or more items [23–26]. This was due to a patient selection bias in 4 of the 5 cases. Two studies evaluated non-consecutive samples of AVS patients [25, 27], and two studies reported on selected subsets of AVS patients (strokes in the inferior cerebellar peduncle [24] or AVS with AUHL [23]). The remaining studies (*n* = 7/11) examined relatively unselected patient populations (i.e., isolated or non-isolated AVS with consecutive recruitment, resulting in a low risk for bias of patient selection in the QUADAS2-rating) [10, 26, 28–32]. The final study with high risk of bias (in this case, with respect to the reference standard) did not obtain the reference test (delayed MRI-DWI) in all patients with peripheral AVS (pAVS) [26].

### Characteristics of studies and patients

Of the 14 included articles, three were preliminary reports or selected subcategories from the same study cohorts published [10] and were thus not considered for the quantitative analysis to avoid duplication [9, 33, 34]. The study design was prospective in 8/11 studies (*n* = 635 patients) and retrospective in 3/11 studies (*n* = 135 patients). In most studies patients were examined either by expert neuro-otologists (*n* = 8 studies, *n* = 538/770 [69.9%] patients) or trained non-subspecialists (i.e., trained neurology residents, *n* = 2 studies, *n* = 138/770 [17.9%] patients [28, 30]), while in one study they were assessed by untrained non-subspecialists (i.e., neurology residents without dedicated neuro-otology training, *n* = 94/770 [12.2%] patients [31]). Ten out of 11 studies reported on both peripheral and central lesions, while the remaining study included only cases with inferior cerebellar peduncle lesions [24]. The proportion of central AVS cases was 48.7% (*n* = 375/770). Ischemic stroke was the most common central cause (*n* = 319/375 [85.1%]), with a predominance of PICA-territory strokes (*n* = 173/270 [64.1%] among those with territory reported). Acute unilateral vestibulopathy (i.e., vestibular neuritis [without hearing loss, *n* = 385] or labyrinthitis [with hearing loss, *n* = 6]) was diagnosed in all but four patients with peripheral AVS (pAVS; *n* = 391/395 [99.0%]) (see Table 1 for details).

**Table 1:** Key epidemiologic aspects

	Number of studies	cAVS patients (*n*)	pAVS patients (*n*)
Age (range of mean ages ± 1SD) [years]	11 [10, 23–32]	53.6 ± 21.4 to 63.0 ± 13.8	43.3 ± 14.9 to 64.4 ± 10.9
Gender			
Females	7 [10, 24, 25, 27–29, 31]	113	113
Males	8 [10, 23–25, 27–29, 31]	166	135
Not reported	3 [26, 30, 32]	96	147
Central etiology			
Ischemic	11 [10, 23–32]	319	N/A
PICA	8 [10, 24–27, 29, 30, 32]	173	N/A
AICA	7 [10, 23, 25–27, 29, 30]	45	N/A
SCA	5 [10, 25, 26, 29, 30]	15	N/A
Brainstem	3 [10, 26, 29]	37	N/A
Non-specified vascular territory*	5 [26, 28, 30–32]	49*	N/A
Hemorrhagic	3 [10, 24, 26]	12	N/A
Other central^†^	6 [10, 23, 24, 30–32]	44	N/A
All	11 [10, 23–32]	375	N/A
Peripheral etiology			
Vestibular neuritis	10 [10, 23, 25–32]	N/A	385
Labyrinthitis	2 [10, 32]	N/A	6
Other peripheral^‡^	1 [23]	N/A	4
All	10 [10, 23, 25–32]	N/A	395
Clinical presentation—isolated AVS vs. non-isolated AVS			
Both isolated AVS and non-isolated AVS	7 [10, 23, 25, 26, 29, 30, 32]	274	N/A
Isolated AVS only	4 [24, 27, 28, 31]	101	N/A

	Number of studies	Number of patients (pAVS and cAVS pooled)	
Study design			
Prospective	8 [10, 25, 26, 28–32]	635	
Retrospective	3 [23, 24, 27]	135	
Training background of examiner			
Trained non-specialists (neurologists, neurology-residents)	2 [27, 28]	138	
Non-specialists, non-trained	1 [31]	94	
Experienced neuro-otologists	8 [10, 23–26, 29, 30, 32]	538	
Bedside vestibular testing performed			
HINTS+ battery	11 [10, 23–32]	770	
Vertical or torsional SN	5 [10, 23, 24, 26, 28]	333	
Bedside audiological testing			
Finger rubbing	5 [10, 23, 25, 28, 29]	311	
Whispered words	2 [30, 31]	169	
Tuning fork	1 [27]	114	
Tuning fork and whispered words	1 [26]	97	
Asked about new-onset HL	1 [32]	71	
Quantitative audiologic testing (PTA)			
PTA only	1 [24]	8	
PTA in all patients	3 [23, 27, 32]	198	
Additional PTA (in selected patients)^§^	5 [10, 25, 26, 28, 31]	12	
No PTA	2 [29, 30]	105	

*AICA* anterior inferior cerebellar artery, *APV* acute peripheral vestibulopathy, *AVS* acute vestibular syndrome, *C* central, *cAVS* central acute vestibular syndrome, *HINTS* head impulse test, nystagmus examination, test of Skew, *HINTS+* HINTS plus new unilateral hearing loss, *HL* hearing loss, *iAVS* isolated acute vestibular syndrome, *MRI* magnetic resonance imaging, *N/A* not available, *niAVS* non-isolated acute vestibular syndrome, *P* peripheral, *pAVS* peripheral acute vestibular syndrome, *PICA* posterior inferior cerebellar artery, *PTA* pure tone audiogram, *SCA* superior cerebellar artery, *SN* spontaneous nystagmus

^*^This included nine MR-negative strokes [32]

^†^Other central lesions include multiple sclerosis (*n* = 8), paraneoplastic (*n* = 2), vestibular migraine (*n* = 39), Wernicke encephalopathy (*n* = 1), brainstem encephalitis (*n* = 2), carbamazepine intoxication (*n* = 1), metastatic breast cancer (*n* = 1)

^‡^Other peripheral cases include heavy/light cupula syndrome (*n* = 4)

^§^In two studies numbers for PTA were not reported [10, 31]

### Bedside neuro-otologic examination

**Fig. 2:**
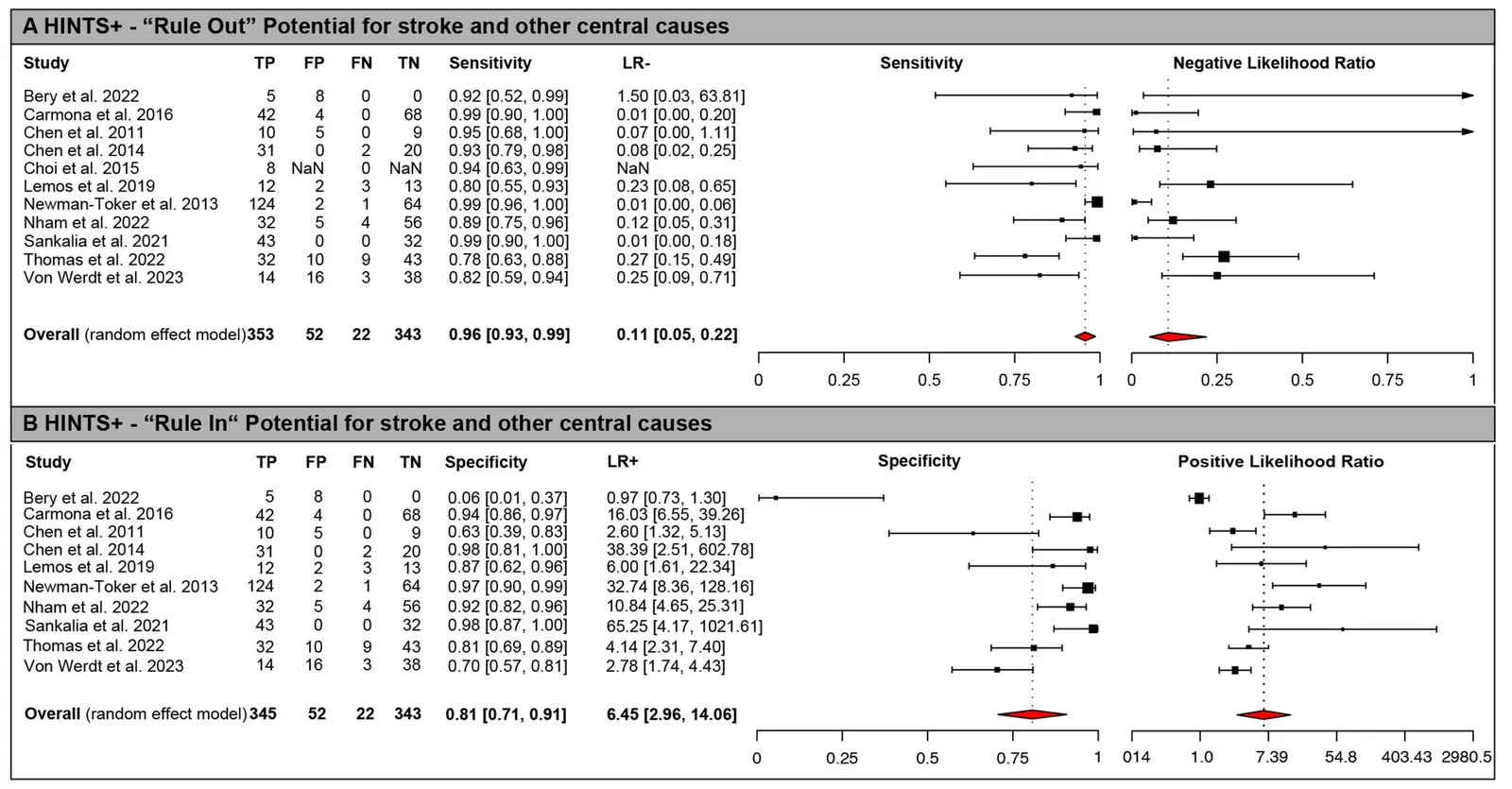
Forest plots of diagnostic test properties for the HINTS+ [10] bedside examination. Panel **A**—sensitivity and negative likelihood ratio (LR−) including 95% confidence intervals (CI); Panel **B**—specificity and positive likelihood ratio (LR+) including 95% CI. Single study results and aggregated values for all test properties (including 95% CI) are provided for all studies included (*n* = 11). Heterogeneity amongst all studies was significant both for ruling out (i.e., sensitivity, *p* = 0.013) and for ruling in (i.e., specificity, *p* < 0.001) central (mostly vertebrobasilar ischemic stroke) causes using Cochran’s *Q*. Note that the axis for positive likelihood ratio uses an exponential scale

All patients underwent HINTS+ testing at the bedside. Bedside HIT alone had moderate diagnostic accuracy (sensitivity = 75.7% [95% CI 65.6–85.8%]; specificity = 95.7% [95% CI 92.6–98.8%]), with 66 cAVS cases having abnormal (“pseudo-peripheral”) HIT (see Table 2). Adding testing for impaired eccentric gaze-holding (gaze-evoked nystagmus, GEN) and skew deviation (i.e., obtaining all three components of the HINTS) increased sensitivity substantially (92.7% [95% CI 88.7–96.8%]), while specificity decreased only slightly (91.1% [95% CI 86.3–95.8%]). By adding AUHL (i.e., HINTS+), sensitivity increased further (95.7% [95% CI 92.7–98.7%)]), but specificity dropped to 80.7% (95% CI 70.6–90.7%) (see Table 2 footnote for impact of bias on specificity). Despite the lower specificity, the loss of diagnostic power for positively identifying a central cause was only modest and not statistically significant in this sample (LR+ 7.15 [HINTS] vs. 6.45 [HINTS+], *p* = 0.28). The Cochran’s *Q* test for heterogeneity among sensitivities and among specificities was both significant with a *p*-value for sensitivity of 0.013 and a *p*-value for specificity of < 0.001 (see Fig. 2).

**Table 2:** Bedside diagnostic accuracy—comparison of different tests

Finding on diagnostic test*	*n* (cAVS)	*n* (pAVS)	Sensitivity % (95% CI)	LR− (95% CI)	Specificity % (95% CI)	LR+ (95%)
Normal hHIT (all)	375	395	75.7 (65.6–85.8)	0.27 (0.16–0.46)	95.7 (92.6–98.8)	11.42 (4.69–27.8)
PICA stroke vs. APV only	173	395	94.8 (90.4–97.2)	0.05 (0.03–0.10)	98.9 (96.7–99.6)	84.05 (27.27–259.09)
AICA stroke vs. APV only	45	395	40.0 (27.0–54.5)	0.62 (0.49–0.79)	97.1 (94.3–98.5)	13.70 (6.34–29.61)
Direction-changing Nystagmus^§^ (all)	373	393	29.5 (20.3–38.8)	0.70 (0.62–0.80)	98.5 (96.9–100.0)	10.18 (3.45–30.05)
Skew deviation (all)	371	393	26.9 (16.1–37.7)	0.76 (0.66–0.87)	97.0 (95.3–98-7)	5.80 (3.44–9.80)
Hearing loss (all)	375	395	11.4 (5.1–17.8)	0.97 (0.94–1.00)	89.6 (83.9–95.3)	1.53 (0.85–2.78)
PICA stroke vs. APV only	173	395	1.2 (0.3–4.1)	1.00 (0.98–1.02)	98.9 (96.7–99.6)	1.03 (0.17–6.07)
AICA stroke vs. APV only	45	395	44.4 (30.9–58.8)	0.58 (0.45–0.75)	96.0 (93.0–97.7)	11.07 (5.70–21.52)
HINTS central (all)	375	395	92.7 (88.7–96.8)	0.14 (0.07–0.28)	91.1 (86.3–95.8)	7.15 (3.76–13.59)
PICA stroke vs. APV only	173	395	98.8 (95.9–99.7)	0.01 (0.00–0.05)	95.1 (91.8–97.1)	20.23 (11.90–34.37)
AICA stroke vs. APV only	45	395	73.3 (59.0–84.0)	0.29 (0.18–0.46)	93.4 (89.9–95.8)	11.16 (6.91–18.04)
HINTS+ central (all)^†^	375	395	95.7 (92.7–98.7)	0.11 (0.05–0.22)	80.7 (70.6–90.7)^†^	6.45 (2.96–14.06)
PICA stroke vs. APV only	173	395	98.8 (95.9–99.7)	0.01 (0.00–0.05)	95.1 (91.8–97.1)	20.23 (11.90–34.37)
AICA stroke vs. APV only	45	395	91.1 (79.3–96.5)	0.10 (0.04–0.25)	92.3 (88.6–94.9)	11.89 (7.80–18.11)

*AICA* anterior inferior cerebellar artery, *APV* acute peripheral vestibulopathy, *cAVS* central acute vestibular syndrome, *CI* confidence interval, *hHIT* horizontal head-impulse test, *HINTS* head impulse test, nystagmus examination, test of Skew, *HINTS+* HINTS plus new unilateral hearing loss, *LR−* negative likelihood ratio, *LR+* positive likelihood ratio, *pAVS* peripheral acute vestibular syndrome, *PICA* posterior inferior cerebellar artery

^*^Analyses included both central and peripheral cases. For each test, the bolded row is denoted as “(all)” to reflect the fact that all patients from studies reporting on that test are included. In the indented rows that follow most of the tests, we report on the subsets of ischemic strokes restricted to either the PICA or AICA territory; not all studies reported vascular territory at the patient level, so these central n’s do not sum to the total

^†^Two out of 11 studies included more selective study populations (i.e., high risk of bias in patient selection), with either enrichment of pAVS cases with AUHL [23], or cAVS cases with a specific lesion location (inferior cerebellar peduncle) [24]. These studies may have biased estimated specificity downward, whereas this was unlikely the case with the other two studies that had a high risk-of-bias for patient selection due to nonconsecutive AVS samples [25, 27]. Thus, calculations were repeated excluding those two studies with highly selected subgroups [23, 24], which resulted in a substantially smaller drop in specificity when comparing HINTS and HINTS+. Whereas the drop in specificity was 10.4% when including all studies (91.1% [95% CI 86.3–95.8%] vs. 80.7% [95% CI 70.6–90.7%]), it was only 0.4% when focusing on studies that included relatively unselected study populations (91.1% [95% CI 86.3–95.8%] vs. 90.7 [95% CI 85.6–95.8%]). Likewise, whereas the LR+ decreased (i.e., reduced diagnostic “rule in” power) from 7.15 (3.76–13.59) to 6.45 (2.96–14.06) when applying the HINTS+ and including all studies, we noted a slight increase in LR+ (i.e., greater diagnostic “rule in” power) from 7.15 (3.76–13.59) to 7.62 (4.04–14.30) for HINTS+ when excluding these two studies. When applying HINTS+ (and including all studies), a decrease in LR- (i.e., greater diagnostic “rule out” power) from 0.14 (0.07–0.28) to 0.11 (0.05–0.22) was observed, whereas LR- for HINTS+ was 0.10 (0.05–0.20) when removing those two studies, indicating a slightly larger decrease (again, even greater “rule out” power). Note also that the aggregate specificity for HINTS+ for all strokes is well below that estimated for either the PICA or AICA subgroup; this related issue is also attributed to the significant heterogeneity across studies, including patient selection

A sensitivity analysis was performed, comparing the diagnostic accuracy of HINTS+ when including all 11 studies vs. only those 9 studies that included patients from relatively unselected patient populations [10, 25–32]. Thereby, no significant differences in diagnostic accuracy parameters were noted when removing those two studies with highly-selected patient populations [i.e., non-significant differences in *p*-values for sensitivity (*p* = 1.000), specificity (*p* = 0.448), LR− (*p* = 0.960), and LR+ (*p* = 0.454)] [23, 24].

### The impact of lesion location on diagnostic accuracy in cAVS

Sensitivity for detecting a central (ischemic) lesion using the HIT alone was much higher for PICA strokes than for AICA strokes (94.8% [95% CI 90.4–97.2%] vs 40.0% [95% CI 27.0–54.5%], *p* < 0.001, Fisher’s exact test) (see Table 2). Including all three components of HINTS increased sensitivity only slightly for PICA strokes, whereas a substantial gain was seen for AICA strokes (PICA 98.8% [95% CI 95.9–99.7%] vs. AICA 73.3% [95% CI 59.0–84.0%], *p* < 0.001). When adding the fourth sign (AUHL), HINTS+ sensitivity for PICA strokes remained unchanged (relative to HINTS), while sensitivity increased substantially for AICA strokes, though a difference still remained (PICA 98.8% [95% CI 95.9–99.7%] vs. AICA 91.1% [95% CI 79.3–96.5%], *p* = 0.018). Specificities of HIT, HINTS, and HINTS+ were not significantly different between PICA vs. AICA strokes (*p* > 0.05). Notably, specificities for HINTS+ for both AICA and PICA subgroups were meaningfully higher (95.1% and 92.3%, respectively) relative to the overall specificity of HINTS+ across all cases (80.7%) (Table 2); this was attributed to heterogeneity across studies.

### The frequency and pattern of unilateral new-onset hearing loss in AVS

Hearing was assessed at the bedside by finger rub (*n* = 311) [10, 23, 25, 28, 29], tuning fork tests (*n* = 114) [27], whispered words (*n* = 169) [30, 31], both finger rub and whispered words (*n* = 97) [26] or by bedside pure tone audiometry (*n* = 71) [32] (see Table 3). In one study, AUHL was an exclusion criterion for pAVS cases [26], and in one study only hearing loss as reported by the patient was considered for bedside assessment [32].

**Table 3:** Assessment of hearing loss and related vestibular deficits

	cAVS		pAVS*	
Diagnostic test	Affected	All	Affected	All
New-onset unilateral hearing loss^†^	29^†^	375	21	395
Abnormal bHIT in those with new-onset HL	22	29	15	21
New-onset HL on side with abnormal bHIT	22	22	14	15
New-onset HL ipsilesional to side with acute lesion	29	29	NaN	NaN
Lesion type and vascular territory affected (in case of ischemic lesions) in those with new-onset unilateral HL				
Abnormal HIT ipsilesional to new-onset HL				
AICA	19 [10, 23, 25–27, 30, 32]	45	NaN	NaN
PICA	0	173	NaN	NaN
Brainstem midline perforator	2 [10, 29]	37	NaN	NaN
Focal neurological findings^‡^	3 [32]	9	NaN	NaN
Multiple sclerosis	1 [23]	8	NaN	NaN
Normal HIT ipsilesional to new-onset HL				
AICA	2 [27, 32]	45	NaN	NaN
PICA	2 [10, 29]	173	NaN	NaN

*AICA* anterior inferior cerebellar artery, *bHIT* bedside head-impulse test, *cAVS* central acute vestibular syndrome, *HIT* head-impulse test, *HL* hearing loss, *pAVS* peripheral acute vestibular syndrome, *PICA* posterior inferior cerebellar artery

^*^We maintained definitions as originally used by the authors, even if not consistent with the definitions of the Bárány Society as, for example, for AUVP vs. labyrinthitis [19]

^†^In one study, HL was an inclusion criterion [23]

^‡^In one study, a diagnosis of stroke was based on focal neurological findings only (MR-negative strokes) ipsilesional to the new-onset HL [32]

AUHL was identified in 50 patients (central = 29/375 [7.7%]) vs. peripheral = 21/334 [6.3%] *p* = 0.467 Fisher’s exact; note the peripheral denominator excludes 61 patients from the study, which excluded pAVS cases with AUHL by design) [26]. Diagnostic accuracy for AUHL as a single predictor of stroke was low with a sensitivity of only 11.4% (95% CI 5.1–17.8%), while specificity was relatively high at 89.6% (95% CI 83.9–95.3%). When taking the vascular territory into account, the sensitivity for detecting a stroke was substantially higher for AICA strokes than for PICA strokes (44.4% [95% CI 30.9–58.8%] vs. 1.2% [95% CI 0.3–4.1%], *p* < 0.001), whereas the specificity was not significantly different (*p* = 0.055).

From the 29 cAVS cases with AUHL, vascular lesions were seen in 25 cases [10, 23, 25–27, 29, 30, 32] and an acute demyelinating multiple sclerosis lesion in 1 case [23]. In the remaining three cases, a diagnosis of MRI-negative stroke was made [32]. Among the 21 peripheral cases with AUHL, diagnoses included vestibular neuritis (*n* = 13), inferior-branch vestibular neuritis (*n* = 3); light/heavy cupula syndrome (*n* = 4), and labyrinthitis (*n* = 1).

Bedside HIT was abnormal in 25 of 29 central cases with AUHL, with 19 of these 25 cases due to an AICA stroke. Thus, 90.5% (*n* = 19/21) of all AICA strokes with AUHL had an ipsilaterally abnormal HIT, whereas this was the case in 0% (*n* = 0/2) of PICA strokes with AUHL. Overall, among cAVS patients with abnormal (i.e., peripheral-appearing) HIT (*n* = 66/375 [17.6%]), ipsilesional AUHL was noted in 25 of 66 cases (37.9%).

Pure tone audiometry (PTA) was performed in selected patients in 3 studies (*n* = 12 patients) [25, 26, 28] and in all patients in 4 studies (*n* = 206 patients) [23, 24, 27, 32]. In two studies, the number of patients undergoing PTA was unclear [10, 31]. How hearing loss on PTA was reported varied from study to study. Whereas in some studies only “objective hearing loss” [32] or “severe hearing loss” [28] was noted, others reported an average loss over 3 frequencies (500 Hz, 1000 Hz, and 2000 Hz) [23] or provided detailed hearing loss patterns [25]. However, the majority of studies (5/11) did not provide any detailed information on PTA results or did not include PTA measurements (2/11). The extent of AUHL reported varied from 24 dB (average over 3 frequencies, absolute threshold) to complete deafness.

## Discussion

This meta-analysis confirms and extends prior work demonstrating that the sensitivity of HINTS for detecting stroke or other central causes is slightly increased by adding AUHL (HINTS+), reaching 95.7%. The cost of the 3.0% gain in sensitivity was a decrease in specificity of 10.4% down to 80.7%, although the pooled estimate for lower specificity appears largely attributable to bias in patient selection (see Table 2 footnote); even still, the loss of diagnostic power for positively identifying a central cause was only modest (9.8% from LR+ 7.15–6.45) and not statistically significant. Importantly, adding a search for AUHL had the biggest impact for those diagnosed with AICA strokes, increasing sensitivity for central causes by 17.8% while maintaining high specificity at 92.3%. The proportion of AICA strokes (*n* = 45/218 [20.6%]) was lower in our AVS cohort than PICA strokes, similar to what has been reported previously in large series of cerebellar strokes [35]. These findings support an approach to diagnosis of AVS that incorporates bedside testing of hearing to assess for AUHL, especially in populations where AICA strokes may be prevalent (e.g., older patients with vascular risk factors).

About 10 percent of vertebrobasilar strokes present with AUHL, mostly associated with lesions in the AICA territory [36]. Among the cohort of cAVS patients in our systematic review, those with AICA strokes had a distinct clinical pattern, with frequently combined unilateral AUHL and abnormal HIT ipsilaterally, i.e. audio-vestibular impairment, being observed in 42% (19/45). Although AUHL can, in principle, result from involvement of central auditory structures such as the cochlear nucleus or the acoustic striae [37], in patients presenting with an AVS it most commonly reflects ischemia of the labyrinthine artery. Accordingly, AUHL in AVS is typically associated with combined cochlear and vestibular labyrinthine infarction, and may occur with or without additional cerebellar signs [16]. From a vascular perspective, this pattern can arise either from an isolated labyrinthine artery infarction affecting peripheral structures (the eighth cranial nerve or inner-ear end organs) or from an AICA-territory infarction involving the lateral pons, where the vestibular nerve enters the brainstem and the vestibular and cochlear nuclei lie immediately adjacent to one another [37]. The normal variation in origin for the internal auditory artery (~ 80% AICA, ~ 15–20% basilar, ~ 2–3% PICA) [38], which gives rise to the labyrinthine artery, likely explains the strong association between AUHL and AICA-territory strokes. The potential for hearing loss to accompany basilar-territory ischemia presenting as AVS [39] raises the clinical stakes of using HINTS+ even further. Importantly, isolated labyrinthine infarction may be a harbinger of AICA stroke and emphasizes urgent further workup, as recently reviewed [17].

### Substantial variation in assessing acute-onset unilateral hearing loss at the bedside

Testing of AUHL at the bedside in the studies included in this meta-analysis was non-standardized and examiner-dependent, bearing the risk of both false positive and false negative results. Furthermore, in most studies, no gold standard for assessing hearing loss was available (pure tone audiometry [PTA] was performed in just 28.3% [218/770] of 770 patients). For frontline bedside assessment in emergency medicine, the Society for Academic Emergency Medicine’s GRACE-3 Guideline recommends the use of finger rub [40]. Such a recommendation might be sharpened by distinguishing between conductive hearing loss and sensorineural hearing loss using Weber and Rinne testing, as recommended by a US-based otolaryngology guideline [41].

Relative to PTA, bedside hearing tests such as whispered speech, finger rubbing and fork tests have substantially lower sensitivities, which ranged between 5 and 60% in a head-to-head comparison where PTA served as the reference standard [42]. To address such limitations of bedside screening tests, calibrated finger rubbing (CALFRAST) can be used to detect hearing loss (as it was in one study included in our meta-analysis) [23]. This approach has been demonstrated to be accurate and reliable [43], but still should be confirmed by PTA.

More recently, smartphone apps have been promoted for a self-administered hearing test such as the hearWHO app [44]. Studies evaluating the diagnostic accuracy of an app-based hearing assessment, however, have shown mixed results [45]. While smartphone apps such as hearWHO are promising for early detection of hearing loss, these apps need to be further tested and validated.

### Differential diagnosis in pAVS cases with new-onset unilateral hearing loss

Definitions for the disease terms *vestibular neuritis* and *labyrinthitis* vary, with some sources drawing a clinical distinction (without vs. with auditory symptoms) and others drawing an anatomic distinction (nerve vs. end organ). According to the position paper of the Bárány Society on AUVP, lack of evidence for new-onset hearing loss or tinnitus is required for a diagnosis of AUVP (a.k.a. vestibular neuritis) [19]. In the same position paper, labyrinthitis was defined as a peripheral vestibular disorder associated with ear pain, reduced hearing and/or tinnitus that can demonstrate an acute, subacute, or slowly progressive natural history. This means that, for some patients with combined peripheral audio-vestibular loss (i.e., those without pain), neither term applies. Furthermore, patients with pain plus clear evidence of nerve rather than end-organ involvement (e.g., those with Ramsay Hunt syndrome caused by varicella zoster virus infection) may also be left without a clear vestibular label, since their syndrome fits the recent labyrinthitis definition but not its presumed localization.

#### Different causes of labyrinthine dysfunction—ischemia vs. inflammation

Unilateral new-onset combined audio-vestibular loss with negative delayed MRI-DWI indicates a pathology restricted to the 8th cranial nerve or labyrinth [46]. The middle ear and mastoid should first be inspected for signs of bacterial infection that might have extended to the labyrinth. A careful search for subtle signs such as mild facial weakness or hidden vesicles on the palate, pinna, or external auditory canal that likely suggest varicella zoster virus infection should also routinely be made, especially in those patients with immunosuppression. When in doubt, MRI, including contrast agents and a lumbar puncture, should be considered.

When such signs are absent, even if a viral cause is suspected, labyrinthine infarction should always be considered. The distinction between acute labyrinthine infarction and acute viral labyrinthitis mainly depends on careful history taking and examination [17, 47, 48]. Thereby, the timely dynamics of symptom onset may help in the differential diagnosis. Whereas in AICA stroke the onset of AUHL may be peracute, symptoms may build up more slowly, or staggering in labyrinthitis. Vascular risk factors, accompanying focal neurologic signs and lack of ear pain make a vascular cause more likely, whereas a recent viral upper respiratory tract infection may suggest a viral cause of the acute audio-vestibular loss. Even absent neurological findings, ischemia can result from occlusion of the labyrinthine artery, which is typically a branch of the AICA; thus, cochleo-labyrinthine infarction can be a “peripheral-only” AICA stroke without demonstrable brain stroke. Repeated MR-imaging, including 3D-FLAIR sequences, may demonstrate a high-signal-intensity labyrinthine lesion without gadolinium enhancement, supporting an ischemic cause [49] or hemorrhage [48]. Alternatively, delayed (4 h post contrast) 3D-FLAIR sequences have been shown to identify labyrinthine ischemia in single cases (see [17] for review).

### Limitations

Our study has several limitations. Heterogeneity in bedside methods of detecting hearing loss, which often lacked detailed description, may have impacted our findings. Without pure-tone audiometry as a gold-standard to validate AUHL and no previous (baseline) audiometry for comparison, we cannot compare AUHL detection methods or inter-examiner differences. While in one study the proportion of patients with asymmetric hearing loss > 60 dB on PTA and cAVS was higher [32], there were insufficient data for us to assess whether sudden-onset or more severe hearing loss (contemplated as possible stroke predictors) were signs of central pathology. None of the included studies predefined asymmetrical hearing loss (e.g., an interaural PTA difference threshold) or applied a standardized approach to identifying subtle cochlear deficits. As a result, mild unilateral/asymmetric hearing loss, particularly in central (stroke) cases, may have been missed or dismissed, potentially leading to an underestimation of AUHL in central AVS. Furthermore, the majority of patients included here were examined by expert neuro-otologists, thus the generalizability of our findings for emergency physicians and general neurologists may be limited. However, previously we have shown that the sensitivity of HINTS for detecting a central (ischemic) stroke was similar in both moderately trained non subspecialists and sub-specialists, emphasizing that HINTS(+) can be mastered by non-specialists also [14].

While most studies included relatively unselected patient populations presenting as AVS, two did not [23, 24]; this appears to have lowered estimated specificity due to enrichment of pAVS cases with AUHL. With a fraction of 49% central AVS cases, an enrichment of stroke cases may have biased our results. In Table 4, we provide estimates of how much the post-test probability for stroke using either HINTS or HINTS+ to rule out stroke in the spontaneous acute vestibular syndrome varies depending on the pre-test probability of stroke and the prevalence of AUHL amongst all AVS patients to provide a sense of the potential size of impact. Different reference standards could have impacted our results. One study obtained reference standard MRI-DWI in only a subset of pAVS cases [26], so, theoretically, up to 56/61 cases might have been misclassified as peripheral. Another study permitted a stroke diagnosis based on clinical features despite negative delayed MRI-DWI—three patients had central HINTS+ and received a diagnosis of “minor stroke” despite negative imaging [32]; thus, up to three cases might have been misclassified as central. In several papers, vestibular neuritis cases with AUHL were reported, whereas it remained unclear how this entity was defined. Likely, these patients did not meet the diagnostic criteria of labyrinthitis (i.e., including ear pain) according to the Bárány Society [19]. Likewise, the diagnosis of light/heavy cupula syndrome with associated AUHL [23] remains controversial [50, 51].

**Table 4:** Post-test probability of stroke using HINTS and HINTS+ based on stroke prevalence and frequency of hearing loss in the spontaneous acute vestibular syndrome

		Residual (post-test) probability of stroke after a negative test (i.e., false negatives)		
Prevalence of stroke in AVS population (based on vascular risk profile)	Prevalence of AUHL in AVS population (based on local population distribution of AICA vs. PICA strokes)	MRI-DWI (sens = 81.1; spec = 99.9; LR− = 0.21)* (%)	HINTS (sens = 92.7%; spec = 91.1; LR− = 0.14) (%)	HINTS+ (sens = 95.7%; spec = 80.7; LR− = 0.11) (%)
10% (low)	6.5% (current data)	2.3	1.5	1.2
25% (average)	6.5% (current data)	6.5	4.5	3.5
50% (high)	6.5% (current data)	17.4	12.3	9.9
25% (average)	1% (many fewer AICA strokes)	6.5	4.5^†^	4.3^‡^
25% (average)	6.5% (current data)	6.5	4.5^†^	3.5^‡^
25% (average)	20% (many more AICA strokes)	6.5	4.5^†^	1.6^‡^

*AUHL* acute unilateral hearing loss, *HINTS* head impulse test, nystagmus examination, test of Skew, *HINTS+* HINTS plus new unilateral hearing loss, *LR−* negative likelihood ratio, *LR+* positive likelihood ratio, *sens* sensivitiy, *spec* specificity

^*^For magnetic resonance imaging with diffusion-weighted sequences [MRI-DWI], diagnostic accuracy values used as published by Shah and colleagues were used [52]

^†^Since AUHL is not a component of HINTS (only of HINTS+), varying the prevalence of AUHL in the AVS population will not affect the post-test probability of stroke when applying HINTS

^‡^We systematically varied the prevalence of AUHL among AVS cases, assuming an average probability of stroke in AVS of 25%. With a higher prevalence of AUHL, a larger benefit of using HINTS+ instead of HINTS is apparent, as reflected in the lower post-test probability of stroke with a 20% prevalence of AUHL relative to the higher post-test probability with a 1% prevalence of AUHL in AVS

## Conclusions

In patients fulfilling all criteria of an AVS (including spontaneous nystagmus), use of HINTS+ had very high sensitivity for central causes (95.7%), representing a slight increase (3.0%) over HINTS without AUHL. Adding AUHL had the biggest impact for those with AICA strokes, increasing sensitivity for central causes by 17.8%. The cost of this greater sensitivity was an increase in the false-positive rate from 4.3% (HINTS) to 19.3% (HINTS+) (i.e., a loss of specificity), though this may have been due to bias in patient selection. Even still, the loss of diagnostic power for positively identifying a central cause was modest and not statistically significant in this sample. Clinicians should strongly consider including assessment of AUHL in their routine practice when evaluating patients with new AVS, especially in clinical settings or contexts where AICA strokes are likely to be more prevalent (e.g., among those with substantial vascular risk factors). Care should be taken when interpreting AUHL in the context of known antecedent hearing loss (or HINTS in the context of prior vestibulopathy, for that matter). Future research should focus on the reliability of bedside assessment of AUHL, defining quantitative thresholds for PTA in AVS, and identifying features of hearing loss that may increase specificity for identifying strokes without loss of sensitivity (e.g., sudden-onset or moderate to high-severity hearing loss).

## Supplementary Information

Below is the link to the electronic supplementary material.

Supplementary file1 (DOCX 32 KB)

Supplementary file2 (DOCX 70 KB)

Supplementary file3 (DOCX 47 KB)

## Data Availability

Source data used for the systematic review will be made available to others upon request to the corresponding author.
