# Supplementary material for: HINTS+ to diagnose AICA stroke: systematic review of the diagnostic impact of acute unilateral hearing loss in the acute vestibular syndrome
Source: J Neurol. 2026 Feb 10;273(2):131. doi: 10.1007/s00415-026-13641-3 (PMC13668945; doi:10.1007/s00415-026-13641-3)
Supplement: Supplementary file 1 [file 415_2026_13641_MOESM1_ESM.docx]

# **Appendix 1- electronic search strategy, coding-scheme for the systematic review and data analysis**

**The search strategy was designed by a neurologist with relevant domain expertise in neuro-otology (AAT). We searched MEDLINE and Embase for English-language articles, using the following strategies with the following components: (1) vertigo/dizziness, (2) diagnostic accuracy of of HINTS+ as assessed at the bedside, and (3) acute vestibular syndrome (ischemic stroke, acute peripheral vestibulopathy). We also performed a manual search of reference lists from eligible articles and contacted corresponding authors where necessary. We did not seek to identify research abstracts from meeting proceedings or unpublished studies.**

AGGREGATED/COMPOSITE VERSION (September 23th, 2024) [PubMed 3710 abstracts; EMBASE 3477 abstracts]

((dizz*[tiab] OR vertigo[tiab] OR vestibular[tiab]) AND (prodrom*[tiab] OR diagnos*[tiab] OR manifestation*[tiab] OR clinical feature*[tiab] OR symptom*[tiab] OR “physical examination”[mh] OR “medical history taking”[mh] OR physical exam*[tiab] OR professional competence[mh] OR “sensitivity and specificity”[tiab] OR “sensitivity and specificity”[mh] OR “reproducibility of results”[mh] OR “observer variation”[mh] OR “diagnostic tests, routine”[mh] OR “decision support techniques”[mh] OR “bayes theorem”[mh] OR dizziness/physiopathology[mh] OR vertigo/physiopathology[mh] OR dizziness/diagnosis[mh] OR vertigo/diagnosis[mh]) AND (acute peripheral vestibulopathy[tiab] OR labyrin*[tiab] OR vestibular neuritis[tiab] OR vestibular neuronitis[tiab] OR vestibular syndrome[tiab] OR cerebrovascular[tiab] OR stroke*[tiab] OR cerebellar[tiab] OR hemorrhag*[tiab] OR haemorrhag*[tiab] OR vertebrobasilar insufficiency[tiab] OR TIA[tiab] OR transient ischemic attack[tiab]) NOT (animals[mh] NOT humans[mh]) AND eng[la] AND 2000:2024[dp] NOT review[pt])

DISAGGREGATED VERSION [to demonstrate overall structure]

((dizz*[tiab] OR vertigo[tiab] OR vestibular[tiab])

AND

(prodrom*[tiab] OR diagnos*[tiab] OR manifestation*[tiab] OR clinical feature*[tiab] OR symptom*[tiab] OR “physical examination”[mh] OR “medical history taking”[mh] OR physical exam*[tiab] OR professional competence[mh] OR “sensitivity and specificity”[tiab] OR “sensitivity and specificity”[mh] OR “reproducibility of results”[mh] OR “observer variation”[mh] OR “diagnostic tests, routine”[mh] OR “decision support techniques”[mh] OR “bayes theorem”[mh] OR dizziness/physiopathology[mh] OR vertigo/physiopathology[mh] OR dizziness/diagnosis[mh] OR vertigo/diagnosis[mh])

AND

(acute peripheral vestibulopathy[tiab] OR labyrin*[tiab] OR vestibular neuritis[tiab] OR vestibular neuronitis[tiab] OR vestibular syndrome[tiab] OR cerebrovascular[tiab] OR stroke*[tiab] OR cerebellar[tiab] OR hemorrhag*[tiab] OR haemorrhag*[tiab] OR vertebrobasilar insufficiency[tiab] OR TIA[tiab] OR transient ischemic attack[tiab])

NOT (animals[mh] NOT humans[mh])

AND eng[la]

AND 2000:2024[dp]

NOT review[pt])

## **Inclusion and exclusion rules for abstracts & full-text manuscripts**

**All gathered literature was subject to title/abstract screening by two independent reviewers (AAT/GA). Abstract review coding rules are provided below. Full-text screening was applied to all citations considered eligible or possibly eligible by at least one reviewer. Two independent reviewers (AAT/GA) determined whether full-text manuscripts are eligible and, if not, provided a reason for exclusion (see full-text review coding rules below). Differences were resolved by discussion and consensus. GA and AAT completed a hand search of the reference lists of selected articles for additional citations. For citations identified by hand search, the full process was repeated iteratively until no additional manuscripts were found for inclusion. Inter-rater agreement on full-text inclusion was calculated using Cohen’s kappa [1].**

Our prospectively determined protocol did not explicitly exclude studies of only central or peripheral cases. Accordingly, our primary analysis did not exclude these studies either. However, a sensitivity analysis was performed to assess the potential impact on results of this type of study. Note that these studies only contributed to the aspects of diagnostic test properties relevant to the way the population was defined (e.g. stroke-only studies contributed to the sensitivity of acute unilateral hearing loss for detecting stroke, not to the specificity since there were no non-stroke cases).

## **Abstract review coding rules**

1) Coding status options are “Yes”, “No”, “Maybe”. We will review full text of “Yes” and “Maybe”.

2) Err on the side of “Maybe” if there is doubt about a “No”; this is more conservative.

3) If there is only a title, exclude it only if you feel confident; otherwise code it as “Maybe”.

4) Each "No" should be coded with a reason for exclusion.

5) Reasons for exclusion are listed below 0-7. Go through them in order from 0 to 7 for each abstract, coding the first reason for exclusion only, not multiple reasons for exclusion. Only code "0" for “not English” if you are sure it is “not English”.

6) Two independent raters (AAT/GA) will code reason for exclusion, but we will *not* mandate agreement on exclusion reason at the abstract level.

7) Occasionally an abstract seems inappropriate for another reason. In such cases, code as “other”. There should be few abstracts coded as “other.”

## **Abstract reasons for exclusion**

| 0 | not English | manuscript is not in English |
| --- | --- | --- |
| 1 | no data | review paper; no original patient data |
| 2 | not dizziness | no reasonable prospect that the study includes data about dizziness or vertigo |
| 3 | not acute | no reasonable prospect that the study includes data about *acute* (<72 hours) dizziness or vertigo |
| 4 | not diagnosis | no reasonable prospect that the study includes data about clinical diagnostic accuracy of HINTS+ (or its parts) in acute central (specifically stroke) or peripheral (specifically vestibular neuritis) disorders |
| 5 | <5 cases | fewer than 5 subjects (total participants reported, including cases and controls) |
| 6 | abstract only | only abstract available (from poster presentation or talk at conference) |
| 7 | other | any other reason abstract is not included |

## **Full-text review coding rules**

1) Coding status options are “Yes” or “No”.

2) Each "No" should be coded with a reason for exclusion.

3) Reasons for exclusion are listed below 0-6. Go through them in order from 0 to 6 for each full manuscript, coding the first reason for exclusion only, not multiple reasons for exclusion.

4) Two independent raters (AAT/RA) will code reason for exclusion, and we will mandate agreement on exclusion reason at the manuscript level.

5) Coding differences will be adjudicated or consensus will be developed through dialogue.

## **Full-text reasons for exclusion**

| 0 | not English | manuscript is not in English |
| --- | --- | --- |
| 1 | no data | review paper; no original patient data |
| 2 | not dizziness | the study does not include data about dizziness or vertigo |
| 3 | not acute | the study does not include data about *acute* (<72 hours) dizziness or vertigo obtained during the *acute phase* (<72 hours) of disease |
| 4 | not diagnosis | no reasonable prospect that the study includes data about clinical diagnostic accuracy of HINTS+ (or its parts) in acute central (specifically stroke) or peripheral (specifically vestibular neuritis) disorders |
| 5 | <5 cases | fewer than 5 subjects (total participants reported, including cases and controls) |
| 6 | other | any other reason full text is not included |

## **Search Results**

Our search identified 4388 unique citations, of which 4227 (96.3 %) were excluded at the abstract level (see PRISMA flow chart). We did not demand concordance on reason for abstract exclusion, but, among those abstracts with concordant reasons for exclusion (52.3 %, n=2209), the distribution was as follows: **18.7 % were not about vertigo or dizziness,** 17.6 % **did not include data about acute (<72h) vertigo or dizziness**, **9.2 % were** not **about** clinical diagnostic accuracy**; 5.9 % had no original data, 0.9 % had fewer than 5 subjects studied, and 0.1% were not in English.**

We sought to examine 161 full manuscripts (this included 0 articles identified by hand-search). After initial screening, there were a total of 6 disagreements (3.7 %) about study inclusion for the two reviewers (GA and AAT (search period: 2000-2024), kappa=0.82). These differences were resolved by discussion and – if needed - adjudication by a third reviewer. Overall agreement on reason for exclusion was 81.4 %. We demanded concordance on reason for full-text exclusion and resolved differences by discussion.

At the end of our full-text review, 147 studies were excluded and 14 were considered eligible (see PRISMA flow chart). These eligible studies represented 0.3 % of the total (n=4388). Among all full-text manuscripts excluded (91.3 %), the distribution of reason for exclusion was as follows: were **not about** clinical diagnostic accuracy (86.4 %), **did not include data about acute (<72h) vertigo or dizziness** (5.4 %), contained no original data (3.4 %), **had fewer than 5 subjects studied** (2.7 %), **were not about vertigo or dizziness (1.4%) or were not in English despite an English abstract (0.7%)**. Three studies contained preliminary data and were excluded for the quantitative analysis [2-4], with 11 manuscripts remaining for the quantitative analysis (meta-analysis).

For 8 studies we attempted to contact the first or corresponding author for additional study information. All eight authors responded and provided additional information.

**References**

1.	Cohen J (1960) A coefficient for agreement for nominal scales. Educ Psychol Meas 20:37-46

2.	Kattah JC, Talkad AV, Wang DZ, Hsieh YH, Newman-Toker DE (2009) HINTS to diagnose stroke in the acute vestibular syndrome: three-step bedside oculomotor examination more sensitive than early MRI diffusion-weighted imaging. Stroke 40:3504-3510

3.	Saber Tehrani AS, Kattah JC, Mantokoudis G, Pula JH, Nair D, Blitz A, Ying S, Hanley DF, Zee DS, Newman-Toker DE (2014) Small strokes causing severe vertigo: frequency of false-negative MRIs and nonlacunar mechanisms. Neurology 83:169-173

4.	Kattah JC, Badihian S, Pula JH, Tarnutzer AA, Newman-Toker DE, Zee DS (2020) Ocular lateral deviation with brief removal of visual fixation differentiates central from peripheral vestibular syndrome. J Neurol 267:3763-3772
