# Supplementary material for: HINTS+ to diagnose AICA stroke: systematic review of the diagnostic impact of acute unilateral hearing loss in the acute vestibular syndrome
Source: J Neurol. 2026 Feb 10;273(2):131. doi: 10.1007/s00415-026-13641-3 (PMC13668945; doi:10.1007/s00415-026-13641-3)
Supplement: Supplementary file 2 [file 415_2026_13641_MOESM2_ESM.docx]

## **Appendix 2 - QUADAS-2 assessment of included studies**

For included studies, two independent raters (GA/AAT) assessed the risk of bias or applicability concerns using QUADAS-2 [1] tailored study criteria, resolving disagreements by discussion.

The QUADAS-2 tool for quality rating of diagnostic accuracy studies consists of four core domains (patient selection, index test, reference standard, and flow and timing) [1]. Risk of bias is assessed for all four domains, and applicability is assessed for the first three domains. Thus, seven items per study are assessed to rate quality of evidence. For each item, pre-specified conditions must be met to qualify for “low risk” of bias. The tool’s authors recommend review-specific tailoring of the rating criteria and process [1], which we did for all seven criteria (Box S1-1).

Two authors (GA, AAT) performed the QUADAS-2 rating independently for all studies (n=14) included after full-text search and discrepancies were resolved by discussion. Consensus ratings for those studies that were included (n=14) are provided in Table S1.

### *Box S1-1. Tailored QUADAS-2 rating criteria for key domains of bias or applicability concerns (as previously described in [2]*

- Patient selection (bias): Studies were rated as *unclear* if patients were drawn from a prospective patient registry without further explanation of original selection. Studies were rated as *high* risk if patients were chosen in non-consecutive/non-random fashion or if they studied a known clinical or demographic subgroup in whom test properties of bedside findings were likely overstated (e.g., sensitivity of neurologic findings for stroke if only patients with such findings were investigated) or understated (e.g., eye movement exams applied to incorrect subpopulations of dizziness/vertigo, such as head impulse testing applied to patients with episodic, positional vertigo).
- Patient selection (applicability concerns): Studies were rated as *low* risk if they used clear, accepted definitions for AVS and/or positional EVS (Box S1-2) [3, 4]. Studies were rated as *unclear* if patients were classified but without clear definitions (e.g., said to have AVS, but without defining AVS directly or referencing previously-published criteria). Studies were rated as *high* risk if patients were classified with clear definitions that did not align with accepted definitions.
- Index test (bias): Studies were rated as *low* risk if bedside (index) tests were performed by subspecialists (neuro-ophthalmology, neuro-otology, or other clinicians routinely using vestibular tests [e.g., vestibular physical therapists, vestibular lab technicians]) or trained specialists (e.g., neurologists) or generalists (e.g., ED physicians) where rater skills were shown to be accurate and reliable. Studies were rated *unclear* if tests were performed by untrained specialists (general neurologists or otolaryngologists); specialty trainees (e.g., neurology or otolaryngology residents); if the training of those who performed the bedside examinations was either mixed (e.g., specialists and ED physicians); or if training or masking was not specifically reported. Studies were rated as having *high* risk if more than 20% of tests were performed by generalists who were untrained or whose test performance was of low quality or not assessed.
- Index test (applicability concerns): Studies were rated as *low* risk if bedside test techniques were described in sufficient detail to match accepted standards (e.g., “The HIT was performed by displacing the head laterally 10-20 degrees and then rapidly rotating to the center position.”) or reference was made to using techniques as defined in prior publications with sufficient details (e.g., “The HIT was performed as in Newman-Toker et al., 2008”). Studies were rated as having *high* risk if bedside test techniques were described or demonstrated (e.g., by video) to not match accepted standards or were inconsistently applied. Studies were rated as *unclear* if bedside techniques were not described sufficiently (e.g., “We performed the head impulse test”).
- Reference standard (bias): The risk of bias in the reference standard was determined using rules specific to each diagnosis (see Box S1-3).
- Reference standard (applicability concerns): Studies were rated as *low* risk if standard diagnostic criteria were used to define stroke and/or peripheral vestibular disorders, and these criteria were compatible with existing consensus definitions and guidelines [5-10]. Studies were rated as *unclear* if definitions were not reported. Studies were rated as *high* risk if non-standard definitions were applied.
- Flow and timing (bias): Studies were rated as *low* risk if over 80% of patients underwent the same (or an equivalently valid) reference standard in a timely manner. Studies were rated as *unclear* if details were not reported. Studies were rated as *high* risk if the reference standard varied or timing was not appropriate (e.g., if an MRI reference standard was too early or too late, lowering its sensitivity) in more than 20%.

Abbreviations: AVS – acute vestibular syndrome; ED – emergency department; EVS – episodic vestibular syndrome; HIT – head impulse test; LOE – Level of Evidence

### *Box S1-2. Consensus (ICVD) Definitions for Vestibular Syndromes (i.e., dizziness/vertigo subpopulations)*

- Episodic Vestibular Syndrome (EVS): A clinical syndrome of transient vertigo, dizziness, or unsteadiness lasting seconds to hours, occasionally days, and generally including features suggestive of temporary, short-lived vestibular system dysfunction (e.g., nausea, nystagmus, sudden falls).
- Acute Vestibular Syndrome (AVS): A clinical syndrome of acute-onset, continuous vertigo, dizziness, or unsteadiness lasting days to weeks, and generally including features suggestive of new, ongoing vestibular system dysfunction (e.g., vomiting, nystagmus, postural instability).
- Chronic Vestibular Syndrome (CVS): A clinical syndrome of chronic vertigo, dizziness, or unsteadiness lasting months to years and generally including features suggestive of persistent vestibular system dysfunction (e.g., oscillopsia, nystagmus, gait unsteadiness).

Abbreviations: ICVD – International Classification of Vestibular Disorders

### *Box S1-3. Valid reference standards for central and peripheral vestibular disorders with risk of bias*

- Stroke: Strokes must have been confirmed by neuroimaging to be considered valid. The type of imaging and imaging result determined the QUADAS-2 risk of bias on reference standard. To be considered *low* risk, strokes had to be clinically deemed causal and confirmed to be new/acute lesions by neuroimaging. To be considered causal, rather than incidental, strokes must have been found in a brain region appropriate to the clinical syndrome (brainstem, cerebellum, thalamus, or parieto-insular region if hemispheric). To be confirmed new/acute by neuroimaging, strokes must have been demonstrated by (1) MRI-DWI showing restricted diffusion; (2) clearly new lesion evident by post-onset serial CT or MRI (e.g., no lesion on day #1 but present on day #3); or (3) acute brain hemorrhages or large ischemic strokes clearly evident by CT (e.g., based on demarcation, attenuation, and/or mass effect). Studies that demonstrably fell short of these requirements were rated as *high* risk, whereas studies not providing sufficient detail on neuroimaging were considered of *unclear* risk. If testing protocols were non-uniform across patients, no more than 20% of patients could be in a higher risk category to still maintain the lower risk category for QUADAS-2 status.
- Vestibular neuritis (without hearing loss) or labyrinthitis (with hearing loss): Patients must have had an AVS clinical presentation, a spontaneous or gaze-evoked nystagmus consistent with Alexander’s law, evidence of a unilateral vestibular deficit on quantitative physiologic testing (caloric or quantified head impulse testing), and either no associated (new) neurologic deficits to suggest a central nervous system disorder or negative neuroimaging (by CT or MRI) to be considered valid. The type of imaging and time window from onset of AVS symptoms determined the QUADAS-2 risk of bias on reference standard: *low* risk (MRI-DWI 48 hours to 14 days, inclusive), *unclear* risk (MRI-DWI 0-48 hours), *high* risk (otherwise). If the imaging type or time windows were non-uniform across patients, no more than 20% of patients could be in a higher risk category to still maintain the lower risk category for QUADAS-2 status.

Abbreviations: AVS – acute vestibular syndrome; DWI – diffusion weighted imaging; EVS – episodic vestibular syndrome; ICVD – International Classification of Vestibular Disorders

### *Box S1-4. “Obvious” and “subtle” neurologic signs*

- Obvious neurologic signs: We defined the following neurologic signs as “obvious”: facial palsy, hemisensory loss, crossed sensory loss, dysphagia, dysarthria, limb ataxia, mental status abnormality (e.g., lethargy), hemiparesis, ocular motor paralysis, Horner syndrome, or visual field loss.
- Subtle neurologic signs: We defined the following neurologic signs as “subtle”: central eye movements without limited range of motion (e.g., central spontaneous or gaze-evoked nystagmus with central features [e.g., dominantly vertical or torsional in trajectory], ocular lateropulsion, saccadic smooth pursuit, dysmetric saccades, normal vestibulo-ocular reflexes), new hearing loss, or gait/truncal instability. Hearing loss was included here as it often points to a peripheral lesion localization. The last was considered “subtle” because in the context of AVS, many peripheral vestibular patients have some degree of gait or truncal instability, so it does not uniformly point to a central lesion. These “subtle” signs were permitted in the isolated central AVS (icAVS) subgroup.

| **Table S1. QUADAS-2 quality ratings for included studies (n=14)** | | | | | | | | |  |
| --- | --- | --- | --- | --- | --- | --- | --- | --- | --- |
|  |  | **Risk of Bias** | | | | **Applicability Concerns** | | | |
| **Citation** | **Sample size (n)** | **Patient Selection** | **Index Test** | **Reference Standard** | **Flow & Timing** | **Patient Selection** | **Index Test** | **Reference Standard** | |
| Bery et al. (2022) [11] | 13 | High risk* | Low risk | Low risk | Low risk | Low risk | Low risk | Low risk | |
| Carmona et al. (2016) [12] | 114 | High risk* | Low risk | Unclear risk | Low risk | Low risk | Low risk | Low risk | |
| Chen et al. (2011) [13] | 24 | Low risk | Unclear risk | Low risk | Low risk | Low risk | Low risk | Unclear risk | |
| Chen et al. (2014) [14] | 53 | High risk* | Unclear risk | Low risk | Low risk | Low risk | Low risk | Low risk | |
| Choi et al. (2015) [15] | 8 | High risk* | Low risk | Low risk | Low risk | Low risk | Low risk | Low risk | |
| Kattah et al. (2009) [16] | 101 | Low risk | Low risk | Low risk | Low risk | Low risk | Low risk | Low risk | |
| Kattah et al. (2020) [17] | 151 | High risk* | Low risk | Low risk | Low risk | Low risk | Low risk | Low risk | |
| Lemos et al. (2019) [18] | 30 | Low risk | Low risk | Unclear risk | Low risk | Low risk | Low risk | Low risk | |
| Newman-Toker et al. (2013) [19] | 191 | Low risk | Low risk | Low risk | Low risk | Low risk | Low risk | Low risk | |
| Nham et al. (2022) [20] | 97 | Low risk | Low risk | High risk | Low risk | Low risk | Low risk | High risk | |
| Saber Tehrani et al. (2014) [21] | 15 | High risk* | Low risk | Low risk | Low risk | Low risk | Low risk | Low risk | |
| Sankalia et al. (2021) [22] | 75 | Low risk | Low risk | Unclear risk | Low risk | Low risk | Low risk | Low risk | |
| Thomas et al. (2022) [23] | 94 | Low risk | Low risk | Low risk | Unclear risk | Low risk | Low risk | Low risk | |
| Von Werdt et al. (2023) [24] | 71 | Low risk | Low risk | Low risk | Low risk | Low risk | Low risk | Low risk | |

***** A distinction between enriched populations with higher rates of strokes (e.g., consults for suspected strokes) and those that used a case-control design must be made. For the former there was only a high-risk of bias with respect to positive predictive values (PPV) and negative predictive values (NPV), but not with respect to sensitivity, specificity, positive likelihood ratios (LR+) and negative likelihood ratios (LR-) [14, 17, 21]. In contrast, for the latter ones, there is a high risk-of-bias for all diagnostic test properties [11, 15].

**References**

1.	Whiting PF, Rutjes AW, Westwood ME, Mallett S, Deeks JJ, Reitsma JB, Leeflang MM, Sterne JA, Bossuyt PM, Group Q- (2011) QUADAS-2: a revised tool for the quality assessment of diagnostic accuracy studies. Ann Intern Med 155:529-536

2.	Tarnutzer AA, Gold D, Wang Z, Robinson KA, Kattah JC, Mantokoudis G, Saber Tehrani AS, Zee DS, Edlow JA, Newman-Toker DE (2023) Impact of Clinician Training Background and Stroke Location on Bedside Diagnostic Test Accuracy in the Acute Vestibular Syndrome - A Meta-Analysis. Ann Neurol 94:295-308

3.	Newman-Toker DE, Edlow JA (2015) TiTrATE: A Novel, Evidence-Based Approach to Diagnosing Acute Dizziness and Vertigo. Neurol Clin 33:577-599, viii

4.	Newman-Toker D (2017) Vestibular syndrome definitions for the International Classification of Vestibular Disorders. In: Bárány Classification Committee Meeting on March 10-11, 2017. Berlin, Germany

5.	Bhattacharyya N, Gubbels SP, Schwartz SR, Edlow JA, El-Kashlan H, Fife T, Holmberg JM, Mahoney K, Hollingsworth DB, Roberts R, Seidman MD, Steiner RW, Do BT, Voelker CC, Waguespack RW, Corrigan MD (2017) Clinical Practice Guideline: Benign Paroxysmal Positional Vertigo (Update). Otolaryngol Head Neck Surg 156:S1-S47

6.	Easton JD, Saver JL, Albers GW, Alberts MJ, Chaturvedi S, Feldmann E, Hatsukami TS, Higashida RT, Johnston SC, Kidwell CS, Lutsep HL, Miller E, Sacco RL, American Heart A, American Stroke Association Stroke C, Council on Cardiovascular S, Anesthesia, Council on Cardiovascular R, Intervention, Council on Cardiovascular N, Interdisciplinary Council on Peripheral Vascular D (2009) Definition and evaluation of transient ischemic attack: a scientific statement for healthcare professionals from the American Heart Association/American Stroke Association Stroke Council; Council on Cardiovascular Surgery and Anesthesia; Council on Cardiovascular Radiology and Intervention; Council on Cardiovascular Nursing; and the Interdisciplinary Council on Peripheral Vascular Disease. The American Academy of Neurology affirms the value of this statement as an educational tool for neurologists. Stroke 40:2276-2293

7.	Fife TD, Iverson DJ, Lempert T, Furman JM, Baloh RW, Tusa RJ, Hain TC, Herdman S, Morrow MJ, Gronseth GS, Quality Standards Subcommittee AAoN (2008) Practice parameter: therapies for benign paroxysmal positional vertigo (an evidence-based review): report of the Quality Standards Subcommittee of the American Academy of Neurology. Neurology 70:2067-2074

8.	von Brevern M, Bertholon P, Brandt T, Fife T, Imai T, Nuti D, Newman-Toker D (2015) Benign paroxysmal positional vertigo: Diagnostic criteria. J Vestib Res 25:105-117

9.	Lopez-Escamez JA, Carey J, Chung WH, Goebel JA, Magnusson M, Mandala M, Newman-Toker DE, Strupp M, Suzuki M, Trabalzini F, Bisdorff A, Classification Committee of the Barany S, Japan Society for Equilibrium R, European Academy of O, Neurotology, Equilibrium Committee of the American Academy of O-H, Neck S, Korean Balance S (2015) Diagnostic criteria for Meniere's disease. J Vestib Res 25:1-7

10.	Lempert T, Olesen J, Furman J, Waterston J, Seemungal B, Carey J, Bisdorff A, Versino M, Evers S, Newman-Toker D (2012) Vestibular migraine: diagnostic criteria. J Vestib Res 22:167-172

11.	Bery AK, Chang TP (2022) Positive horizontal-canal head impulse test is not a benign sign for acute vestibular syndrome with hearing loss. Front Neurol 13:941909

12.	Carmona S, Martinez C, Zalazar G, Moro M, Batuecas-Caletrio A, Luis L, Gordon C (2016) The Diagnostic Accuracy of Truncal Ataxia and HINTS as Cardinal Signs for Acute Vestibular Syndrome. Front Neurol 7:125

13.	Chen L, Lee W, Chambers BR, Dewey HM (2011) Diagnostic accuracy of acute vestibular syndrome at the bedside in a stroke unit. J Neurol 258:855-861

14.	Chen L, Todd M, Halmagyi GM, Aw S (2014) Head impulse gain and saccade analysis in pontine-cerebellar stroke and vestibular neuritis. Neurology 83:1513-1522

15.	Choi JH, Seo JD, Choi YR, Kim MJ, Kim HJ, Kim JS, Choi KD (2015) Inferior cerebellar peduncular lesion causes a distinct vestibular syndrome. Eur J Neurol 22:1062-1067

16.	Kattah JC, Talkad AV, Wang DZ, Hsieh YH, Newman-Toker DE (2009) HINTS to diagnose stroke in the acute vestibular syndrome: three-step bedside oculomotor examination more sensitive than early MRI diffusion-weighted imaging. Stroke 40:3504-3510

17.	Kattah JC, Badihian S, Pula JH, Tarnutzer AA, Newman-Toker DE, Zee DS (2020) Ocular lateral deviation with brief removal of visual fixation differentiates central from peripheral vestibular syndrome. J Neurol 267:3763-3772

18.	Lemos J, Martins AI, Duque C, Pimentel S, Nunes C, Goncalves AF (2019) Positional Testing in Acute Vestibular Syndrome: a Transversal and Longitudinal Study. Otol Neurotol 40:e119-e129

19.	Newman-Toker DE, Kerber KA, Hsieh YH, Pula JH, Omron R, Saber Tehrani AS, Mantokoudis G, Hanley DF, Zee DS, Kattah JC (2013) HINTS outperforms ABCD2 to screen for stroke in acute continuous vertigo and dizziness. Acad Emerg Med 20:986-996

20.	Nham B, Reid N, Bein K, Bradshaw AP, McGarvie LA, Argaet EC, Young AS, Watson SR, Halmagyi GM, Black DA, Welgampola MS (2022) Capturing vertigo in the emergency room: three tools to double the rate of diagnosis. J Neurol 269:294-306

21.	Saber Tehrani AS, Kattah JC, Mantokoudis G, Pula JH, Nair D, Blitz A, Ying S, Hanley DF, Zee DS, Newman-Toker DE (2014) Small strokes causing severe vertigo: frequency of false-negative MRIs and nonlacunar mechanisms. Neurology 83:169-173

22.	Sankalia D, Kothari S, Phalgune DS (2021) Diagnosing Stroke in Acute Vertigo: Sensitivity and Specificity of HINTS Battery in Indian Population. Neurol India 69:97-101

23.	Thomas JO, Sharobeam A, Venkat A, Blair C, Ozalp N, Calic Z, Wyllie P, Middleton PM, Welgampola M, Cordato D, Cappelen-Smith C (2022) Video head impulse testing to differentiate vestibular neuritis from posterior circulation stroke in the emergency department: a prospective observational study. BMJ Neurol Open 4:e000284

24.	von Werdt M, Korda A, Zamaro E, Wagner F, Kompis M, Caversaccio MD, Mantokoudis G (2024) The acute vestibular syndrome: prevalence of new hearing loss and its diagnostic value. Eur Arch Otorhinolaryngol 281:1781-1787
