# Supplementary material for: HINTS+ to diagnose AICA stroke: systematic review of the diagnostic impact of acute unilateral hearing loss in the acute vestibular syndrome
Source: J Neurol. 2026 Feb 10;273(2):131. doi: 10.1007/s00415-026-13641-3 (PMC13668945; doi:10.1007/s00415-026-13641-3)
Supplement: Supplementary file 3 [file 415_2026_13641_MOESM3_ESM.docx]

# 1) **Supplemental Source Data & Raw Results**

## **Table S3-1: Studies included in the systematic review**

| **Author (citation)** | **Year** | **Total Dizziness Sample (studied, % females)** | **Definition of total sample / dizzy sample** | **Population (Study Focus)** | **Setting (country)** | **Bedside testing performed by** | **LOE*** | **Data collection (source, analysis)** | **Mean age (standard deviation, range)** | **Special comments** |
| --- | --- | --- | --- | --- | --- | --- | --- | --- | --- | --- |
| Bery et al. [1] | 2022 | NR (13, 31%) | AVS patients with new-onset hearing loss | **AVS with hearing loss** | ED, single academic hospital (Taiwan) | experienced neuro-otologist | 2 | Retrospective (ED population, cross-sectional) | cAVS: 56.2 (15.8, 41-77)  pAVS: 61.4 (5.2, 58-69) | New-onset hearing loss was inclusion criterion. |
| Carmona et al. [2] | 2016 | 1218 (114, 58%) | ED presentation due to “dizziness” | **Isolated AVS** (clinical findings: HINTS vs. truncal ataxia) | ED, single academic hospital (Argentina) | trained neurology residents supervised by experienced neuro-otologists | 1 | Retrospective (ED population, cross-sectional) | cAVS: 57.9 (11, NR)  pAVS: 43.3 (14.9, NR) |  |
| Chen et al. [3] | 2011 | 36 (24, 37%) | ED presentation with „acute prolonged rotatory vertigo associated with nausea and/or vomiting, without other brainstem signs“ | **Isolated AVS** (iAVS) (clinical findings) | ED, single academic hospital (Australia) | neurologists with 4 hours of training in neuro-otology | 2 | Prospective (ED population, cross-sectional) | 64.0 (13, 42-83) |  |
| Chen et al. [4] | 2014 | NR (53, 34%) | ED presentations with “acute prolonged spontaneous vertigo (>24 hours) and gait imbalance consistent with AVS” | **AVS** | ED, single academic hospital (Australia) | experienced neuro-otologist | 4 | Prospective (ED population, cross-sectional) | cAVS: 58.5 (15.8, 24-82)  pAVS: 59.2 (14.5, 37-85) |  |
| Choi et al. [5] | 2015 | NR (8, 63%) | ED presentations of stroke patients with acute dizziness due to inferior cerebellar peduncle lesions | **Isolated AVS** | ED, single academic hospital (South Korea) | experienced neuro-otologist | 4 | Retrospective (ED population, cross-sectional) | cAVS: 53.6 (21.4, 18-83) |  |
| Lemos et al. [6] | 2018 | NR (30, 27%) | „Consecutive AVS patients referred for neurotological assessment by the ER neurology team“ | **AVS** | Single academic hospital (Portugal) | experienced neuro-otologist | 2 | Prospective (ED population, cross-sectional) | cAVS: 56.5 (17.8, NR)  pAVS: 53.5 (16.6, NR) |  |
| Newman-Toker et al. [7]  Preliminary reports in Kattah et al. 2020 [8], Saber-Tehrani et al. 2014 [9], and Kattah et al. 2009 [10] | 2013 | NR (191, 40%) | “Patients with at least 1 hour of acute, persistent, continuous vertigo or dizziness with spontaneous or gaze-evoked nystagmus, plus nausea or vomiting, head motion intolerance, and new gait unsteadiness (i.e., AVS), presenting within 1 week of symptom onset“ | **AVS and > 1 stroke risk factor** (diagnosis, clinical features, imaging) | ED, single academic hospital (USA) | experienced neuro-otologist | 1 | Prospective (ED/hospital admission population, cross-sectional) | 61.0 (NR, 18-92) | Patients with initial negative cMRI underwent repeat cMRI for unexplained signs suggesting brainstem location. Based on the dataset provided by the corresponding author, we included one additional patient that was not considered in the original publication. |
| Nham et al. [11] | 2022 | NR (97, NR) | Patients with acute vertigo presenting to the ED | **AVS** | Single academic hospital (Australia) | experienced neuro-otologist | 1 | Prospective (ED population, cross-sectional) | cAVS: NR  pAVS: NR | 41 cases with AVS due to “other causes” were excluded. Furthermore, 10 AVS cases with “MR-negative stroke” were also excluded from the analysis. |
| Sankalia et al. [12] | 2021 | NR (75, NR) | „…with acute-onset first episode of spontaneous vertigo“ | **AVS** | Single academic hospital (India) | Neurology resident supervised by experienced neuro-otologist | 2 | Prospective (ED population, cross-sectional) | cAVS: NR  pAVS: NR |  |
| Thomas et al. [13] | 2022 | 273 (94, 56%) | “…consecutive patients presenting with AVS … to Liverpool Hospital ED” | **Isolated AVS** | Single academic hospital (Australia) | neurology residents | 2 | Prospective (ED population, cross-sectional) | cAVS: 68.5±10.6 for stroke and 53.7±15.0 for migraine  pAVS: 60.1±14.2 | A subgroup that received various other diagnoses (n=39) was excluded for this analysis |
| Von Werdt et al. [14] | 2023 | 145 (71, NR) | AVS patients with new-onset hearing loss | **AVS with hearing loss** | Single academic hospital (Switzerland) | trained neuro-otology physicians | 2 | Prospective (ED population, cross-sectional) | cAVS: NR  pAVS: NR | Bedside pure tone audiometry performed in all patients |

* Level of evidence was determined as defined by (criteria may be found in Appendix 1).

Abbreviations: AVS = acute vestibular syndrome (vertigo, nystagmus, nausea/vomiting, head-motion intolerance, unsteady gait lasting ≥24h); cCT = cranial computer tomography; cMRI = cranial magnetic resonance imaging; ED = emergency department; NA = not applicable; NR=not reported; VN = vestibular neuritis.

**References**

1.	Bery AK, Chang TP (2022) Positive horizontal-canal head impulse test is not a benign sign for acute vestibular syndrome with hearing loss. Front Neurol 13:941909

2.	Carmona S, Martinez C, Zalazar G, Moro M, Batuecas-Caletrio A, Luis L, Gordon C (2016) The Diagnostic Accuracy of Truncal Ataxia and HINTS as Cardinal Signs for Acute Vestibular Syndrome. Front Neurol 7:125

3.	Chen L, Lee W, Chambers BR, Dewey HM (2011) Diagnostic accuracy of acute vestibular syndrome at the bedside in a stroke unit. J Neurol 258:855-861

4.	Chen L, Todd M, Halmagyi GM, Aw S (2014) Head impulse gain and saccade analysis in pontine-cerebellar stroke and vestibular neuritis. Neurology 83:1513-1522

5.	Choi JH, Seo JD, Choi YR, Kim MJ, Kim HJ, Kim JS, Choi KD (2015) Inferior cerebellar peduncular lesion causes a distinct vestibular syndrome. Eur J Neurol 22:1062-1067

6.	Lemos J, Martins AI, Duque C, Pimentel S, Nunes C, Goncalves AF (2019) Positional Testing in Acute Vestibular Syndrome: a Transversal and Longitudinal Study. Otol Neurotol 40:e119-e129

7.	Newman-Toker DE, Kerber KA, Hsieh YH, Pula JH, Omron R, Saber Tehrani AS, Mantokoudis G, Hanley DF, Zee DS, Kattah JC (2013) HINTS outperforms ABCD2 to screen for stroke in acute continuous vertigo and dizziness. Acad Emerg Med 20:986-996

8.	Kattah JC, Badihian S, Pula JH, Tarnutzer AA, Newman-Toker DE, Zee DS (2020) Ocular lateral deviation with brief removal of visual fixation differentiates central from peripheral vestibular syndrome. J Neurol 267:3763-3772

9.	Saber Tehrani AS, Kattah JC, Mantokoudis G, Pula JH, Nair D, Blitz A, Ying S, Hanley DF, Zee DS, Newman-Toker DE (2014) Small strokes causing severe vertigo: frequency of false-negative MRIs and nonlacunar mechanisms. Neurology 83:169-173

10.	Kattah JC, Talkad AV, Wang DZ, Hsieh YH, Newman-Toker DE (2009) HINTS to diagnose stroke in the acute vestibular syndrome: three-step bedside oculomotor examination more sensitive than early MRI diffusion-weighted imaging. Stroke 40:3504-3510

11.	Nham B, Reid N, Bein K, Bradshaw AP, McGarvie LA, Argaet EC, Young AS, Watson SR, Halmagyi GM, Black DA, Welgampola MS (2022) Capturing vertigo in the emergency room: three tools to double the rate of diagnosis. J Neurol 269:294-306

12.	Sankalia D, Kothari S, Phalgune DS (2021) Diagnosing Stroke in Acute Vertigo: Sensitivity and Specificity of HINTS Battery in Indian Population. Neurol India 69:97-101

13.	Thomas JO, Sharobeam A, Venkat A, Blair C, Ozalp N, Calic Z, Wyllie P, Middleton PM, Welgampola M, Cordato D, Cappelen-Smith C (2022) Video head impulse testing to differentiate vestibular neuritis from posterior circulation stroke in the emergency department: a prospective observational study. BMJ Neurol Open 4:e000284

14.	von Werdt M, Korda A, Zamaro E, Wagner F, Kompis M, Caversaccio MD, Mantokoudis G (2024) The acute vestibular syndrome: prevalence of new hearing loss and its diagnostic value. Eur Arch Otorhinolaryngol 281:1781-1787
